# Laboratory Study Phenomenon of Coal and Gas Outburst Based on a Mid-scale Simulation System

**DOI:** 10.1038/s41598-019-51243-4

**Published:** 2019-10-18

**Authors:** Baisheng Nie, Yankun Ma, Shoutao Hu, Junqing Meng

**Affiliations:** 1 State Key Laboratory of Coal Resources and Safe Mining (China University of Mining & Technology (Beijing)), Beijing, 100083 China; 2 Beijing Key Laboratory for Precise Mining of Intergrown Energy and Resources (China University of Mining & Technology (Beijing)), Beijing, 100083 China; 3 Xinjiang Institute of Engineering, Urumqi, 830023 China; 4 Beijing Academy of Safety Engineering and Technology, Beijing Institute of Petrochemical Technology, Beijing, 102617 China

**Keywords:** Natural hazards, Geochemistry

## Abstract

Outburst simulation experiments facilitate understanding coal and gas outburst in underground mining. With the help of the mid-scale simulation system, a model based on similitude principle, coal seam sandwiched by roof and floor, was constructed to conduct an outburst experiment. It had a three-dimensional size of 1500 mm × 600 mm × 1000 mm with 0.5 MPa gas pressure. The experimental procedures include specimen preparation, moulding, sealing, gas charging and adsorption, and completion. The outburst process was investigated by analyzing the gas pressure variation, temperature variation, outburst propagation velocity, particle size of outburst coal and energy transformation. During the experiment, each gas charging was accompanied with gas pressure or temperature fluctuation because of coal behavior of gas adsorption-desorption. The outburst propagation velocity was 17.2 m/s, obtained by a mass-weighted calculation of velocities of outburst coal. The small-size coal particles have a higher desorption rate and tend to participate in outburst process. According to energy conservation law, the energy forms of the outburst included elastic strain energy (*E*_*e*_), gas expansion energy *(E*_*p*_), internal energy of coal (*ΔU*), breakage work (*W*_1_), throwing out work (*W*_2_) and gas-flow loss energy (*ΔE*), and each was calculated respectively. Gas potential energy, including gas expansion energy and internal energy of coal, registered a larger percent and was far greater than the strain energy. And it can be the main factor influencing the occurrence of low-threshold outburst. The experimental system provides a feasible way to study the initiation and evolution of coal and gas outbursts.

## Introduction

In the process of coal exploitation, coal and gas outburst (hereinafter referred to as “outburst”) is a dynamic disaster without any conspicuous precursor. It may occur in workface, with large amount of coal and gas ejecting instantaneously, sometimes leading to large fatalities^1,2^. Outburst can be more frequent as the mining goes deeper^3^. Factors influencing the outburst occurrence and the mechanism of outburst initiation and evolution have not been fully understood^4,5^. Outburst simulation experiment can be an efficient method to carried out further research. Many scholars developed different experimental apparatuses to study outburst phenomenon and made a certain progress.

A shock-tube apparatus was built at Peking University to simulate the sudden decompression of coal samples. Guan *et al*.^6^ hypothesized that the mechanism of outbursts is similar to magma fragmentation during explosive volcanic eruption. Wang *et al*.^7^ used a self-developed shock tube to investigate the energetic failure of gassy coal induced by rapid decompression and desorption. The outburst pipe was designed and outburst experiments on the influence of sorption process were carried out at laboratory conditions^8,9^. Skoczylas^9^ analyzed the impact of uniaxial strength and gas pressure for the outburst to estimate the risk of hazard area. Jiang *et al*.^10^ simulated the outburst induced by rock cross-cut coal uncovering with one dimensional outburst simulation and proposed the “spherical shell losing stability” model. Wang *et al*.^11^ analyzed the contributions of *in situ* stress and gas pressure to the outburst process, using the test system of one-dimensional outburst.

An experimental apparatus was constructed by Tu *et al*.^5^ to simulate the outburst related to gas-rich areas, and the tension effect of gas on coal was observed. Yin *et al*.^12^ developed a comprehensive simulation device with large-sized coal samples. It satisfied the outburst experiments with non-uniform distribution of loadings, a certain size of outburst port and 2 MPa gas pressure^12,13^. Jin *et al*.^14^ developed an apparatus, composed of outburst chamber, simulated roadway, decompression device, data acquisition system and vacuum/gas injection equipment, to study the formation and transport of outburst coal-gas flow in underground roadway and concluded that rapid gas desorption played a decisive role on the promotion of outburst. The outburst simulation system based on CSIRO model was developed by Yuan *et al*.^3^ and test results showed that the coal strength prevented the outburst^15^.

A Mid-scale simulation system for outburst experiment developed by Nie *et al*.^16^ can accommodate specimen with dimensions of 1500 mm × 600 mm × 1000 mm, simulating the coal seam sandwiched by roof and floor and local stress concentration ahead of work face. It made the specimen approximate to coal mining circumstance to a certain degree, and the experiment be flexible in operations. Based on the Mid-scale simulation system, this paper conducted the investigation on the outburst occurrence and propagation by analyzing the variation of gas pressure and temperature, velocity attenuation of outburst coal and distribution of pulverized coal. Additionally, energy conservation law was used to calculate and analyze energy transformation during an outburst. This work may help understanding of the outburst mechanism and provide some implications on outburst prevention in underground mining.

## Methodology

### Experimental system of outburst simulation

#### Simulation model

The similitude-based scaling is often too restrictive because it may not satisfy all of the resulting scaling laws exactly^17^. The notion of approximate similitude is necessary in conducting a research, as exact similitude is impossible^17,18^. The important components should be scaled and the less important ones can be neglected. Coal mass is complex porous and organic rocks. The gas transportation and storage in coal are complex as well. Raw coal sampled from coal workface is used for experiment without additional processing. So certain similar scales about coal materials are set as one, such as adsorption constant, porosity and density. Many scholars adopted one as gas pressure similar scale to conduct outburst simulation^5,11,12,15^. According to similitude principle on solid mechanics, similar conditions are shown as follows^19^.

1 $$\{\begin{array}{c}{C}_{\sigma }={C}_{\gamma }\cdot {C}_{l}\\ {C}_{\sigma }={C}_{E}\,\\ {C}_{p}=1\,\end{array}$$

where *C*_*σ*_*, C*_*γ*_*, C*_*l*_*, C*_*E*_*, C*_*p*_ represent the similarity constants for stress (*σ*), unit weight (γ), geometry size (*l*), Young’s modulus (*E*), gas pressure (*p*). Similarity constant *C*_*i*_ is defined as the ratio of the physical quantity of prototype *i*_*p*_ to that of the model *i*_*m*_, that is:

2 $${C}_{i}=\frac{{i}_{p}}{{i}_{m}}$$

**Figure 1:**
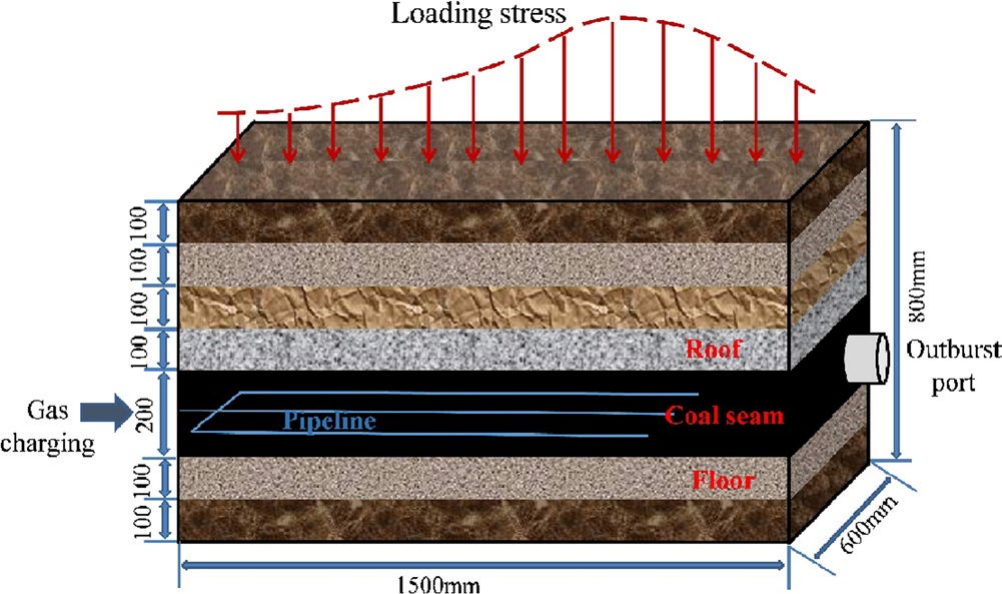
The design idea of outburst simulation.

The experimental model of outburst is shown in Fig. 1. Based on similitude principle, geomechanical model is laid out by preparing similar materials in the experimental apparatus, manifesting outburst-induced factors in coal mining circumstances, such as strata distribution, stress distribution in front of workface, gas pressure and gas content.

#### Simulation system

**Figure 2:**
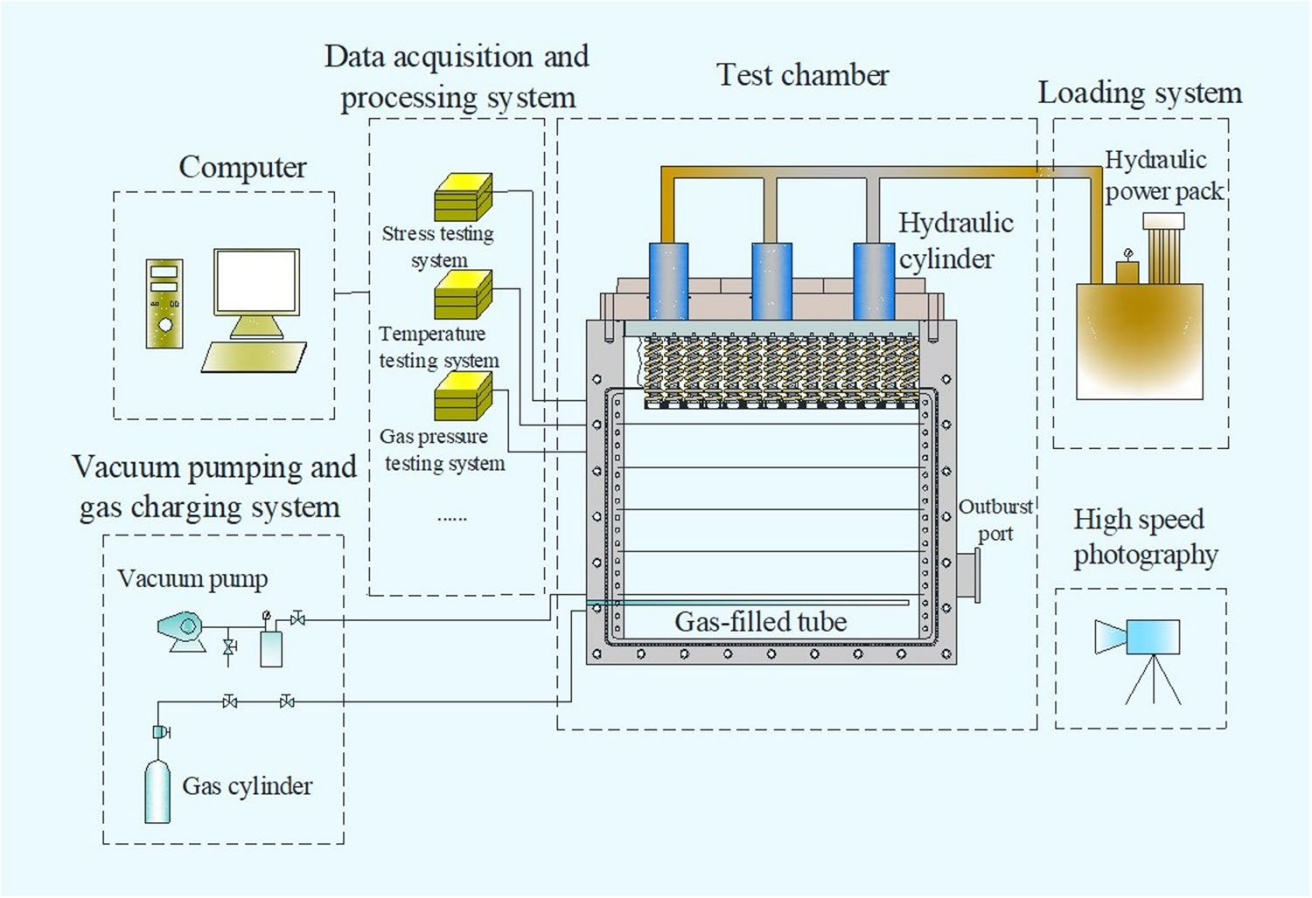
Structure diagram of the outburst simulation system.

**Figure 3:**
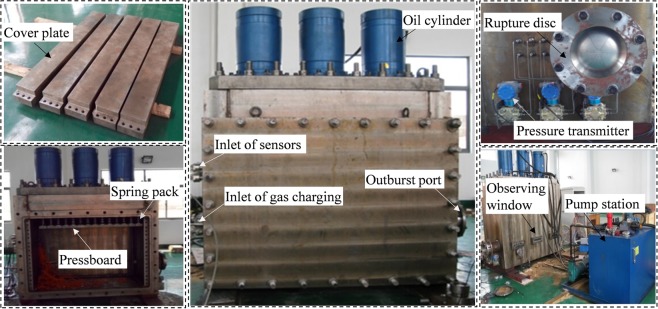
The experimental apparatus.

The mid-scale outburst simulation system consists of test chamber, distributed loading system, outburst-inducing device, vacuum pumping and gas charging system, and data acquisition system (Fig. 2). The physical picture of the experimental apparatus is shown in Fig. 3. Inlets of gas charging and sensors are at the left side, and an outburst port for emitting coal and gas at the right side. Sealing rings of front cover plate make sure good pressure tightness, ranging −0.1~2.0 MPa.

Distributed loading system consists of pump station, oil cylinders, spring packs and pressboards. Maximum pressure supplied by hydraulic power unit is 20.0 MPa. Spring packs connecting pressboards transfer loading stress from oil cylinders. The preset spring packs with varied elastic force distribute loading stress on specimen (Fig. 1) and the concentration factor is 1.6, to achieve non-uniformly distributed loading.

A rupture disc installed on the outburst port is chosen as outburst-inducing device, which is destroyed immediately if the pressure exceeds its threshold value. Vacuum pumping and gas charging system consists of the vacuum gage and pump, purging valve, piezometer, and pipeline. A real-time data acquisition system, including sensors and high-speed photography, collects pressure, temperature, and dynamic phenomenon of outburst coal.

### Experimental procedures

**Figure 4:**
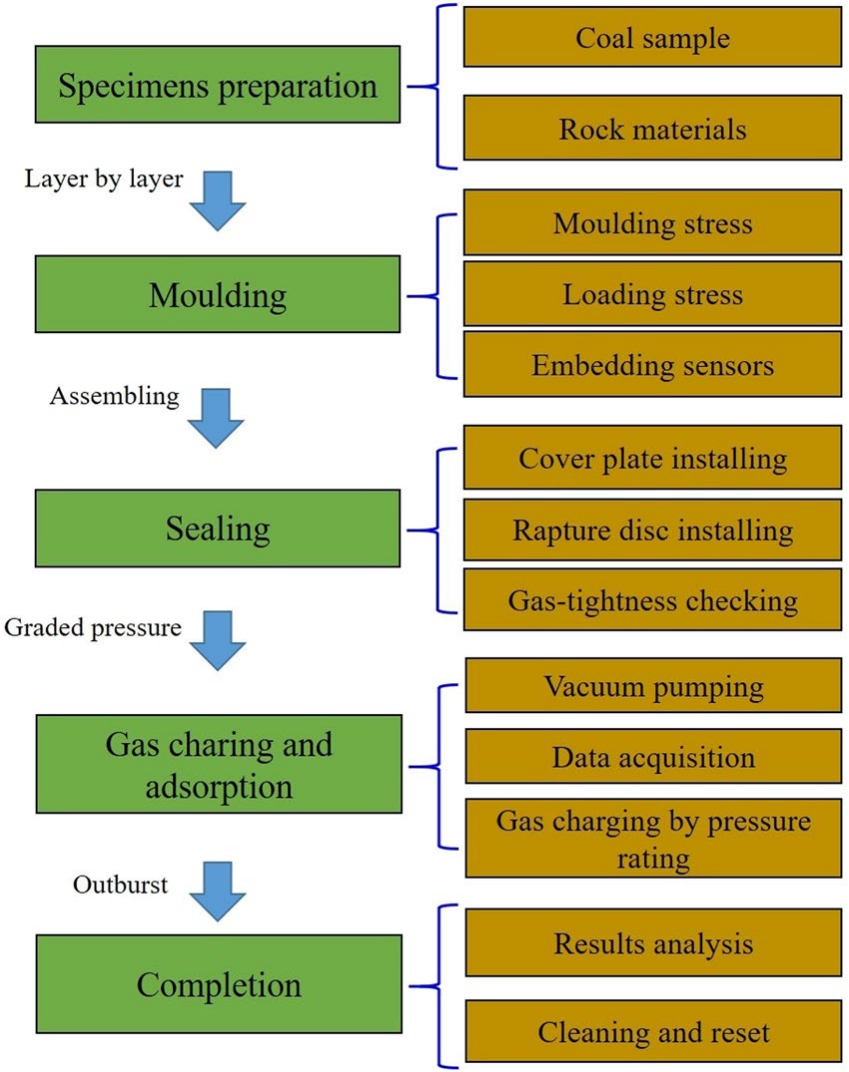
Flow chart of coal and gas outbursts’ test procedures.

The outburst experiment at middle scale is conducted in an open space. The experimental procedures include specimen preparation, moulding, sealing, gas charging and absorption, and completion, as shown in Fig. 4.

#### Specimens preparation

The outburst-prone coal with 560 m buried depth was sampled from No. 2 coalbed at Dashucun Coal Mine in the north of China. A series of physicochemical parameters of coal, such as parameters for the proximate analysis of coal, were measured and listed in Table 1.

**Table 1:** Parameters of Dashucun coal.

Sample	*H*(m)	*ρ*_1_(g/cm^3^)	*ρ*_2_(g/cm^3^)	*f*	*M*_*ad*_(%)	*A*_*d*_(%)	*V*_*daf*_(%)	*FC*(%)
Dashucun coal	548	1.67	1.42	0.347	1.88	18.19	11.17	80.87

*H* is the burial depth of coal seam*, ρ*_1_ is real density*, ρ*_2_ is apparent density*, f* is protodrakonov strength index*, M*_*ad*_ is moisture content*, A*_*d*_ is ash content*, V*_*daf*_ is volatiles content*, FC* is fixed carbon.

Similitude principle for coal is *C*_*σ*_ = *σ*_*p*_*/σ*_*m*_ = 15/1.5 = 10, *C*_*γ*_ = *γ*_*p*_*/γ*_*m*_ = 1 and *C*_*σ*_ = *l*_*p*_*/l*_*m*_ = 10. The samples, smashed and sieved to less than 1 mm, were moulded with mixing 6% water^4^. The layout model has a size of 1.5 m × 0.6 m × 0.8 m, equipped with 0.2 m floor, 0.2 m coal seam and 0.4 m roof (Fig. 1). The floor included argillaceous siltstone and mudstone layers, at thickness of 0.1 m respectively. The roof included four layers at thickness of 0.1 m each, which are silt mudstone, mudstone, silt mudstone and silt rock layers from top to down. The preparation of model materials for each stratum is shown in Table 2.

**Table 2:** Geometry and mixture ratio parameters.

Lithology		Thick/cm	Density/(g/cm^3^)	UCS/MPa	Ratio
					Sand: Lime: Gypsum
Roof	Silt mudstone	10	1.7	0.28	9: 0.7: 0.3
	Mudstone	10	1.7	0.27	9.1: 0.7: 0.2
	Silt mudstone	10	1.7	0.28	9: 0.7: 0.3
	Silt rock	10	1.7	0.29	8.9: 0.7: 0.4
Coal	No. 2	20	1.42	—	—
Floor	Argillaceous siltstone	10	1.7	0.26	8.9: 0.8: 0.3
	Mudstone	10	1.7	0.27	9.1: 0.7: 0.2

UCS is uniaxial compressive strength.

#### Model layout and system assembling

**Figure 5:**
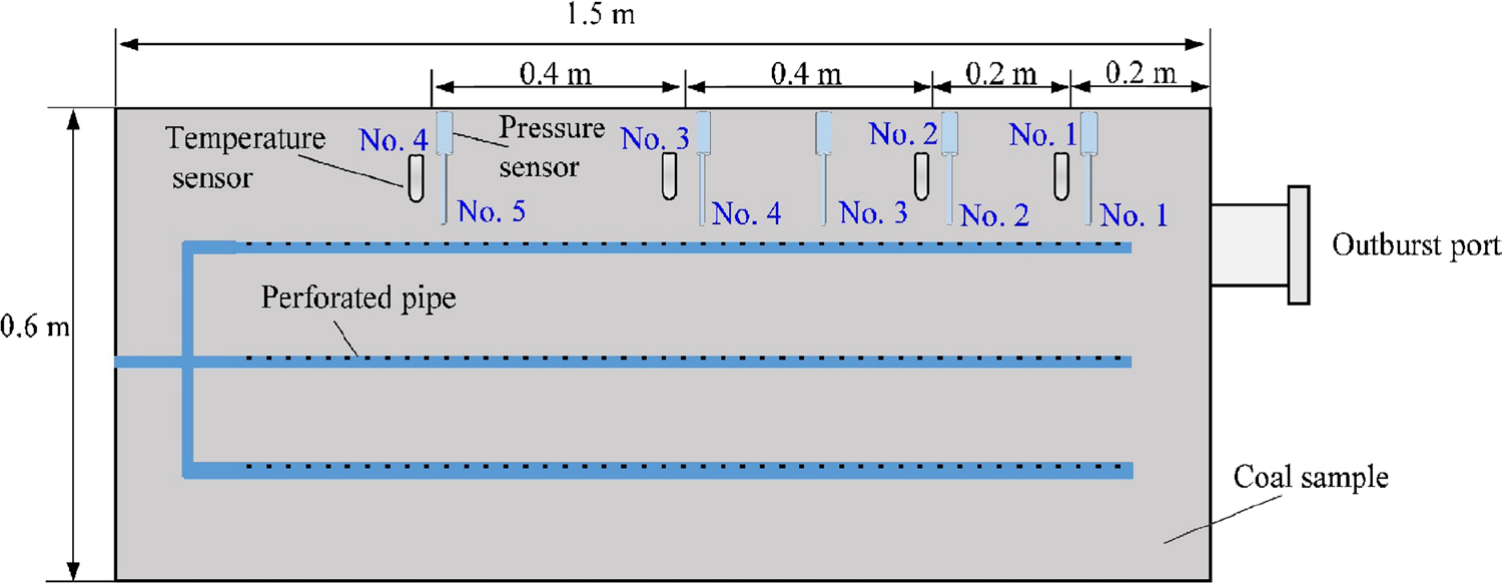
Layout of sensors and pipeline in coal sample (top view).

The geomechanical model was built layer by layer from floor to roof. The materials of each stratum were laid out squarely and moulded with stress of 1.5 MPa for about 30 minutes. The distribution of sensors and pipeline in the model is shown in Fig. 5. Pressure and temperature sensors and pipeline were embedded in coal seam. The metal perforated pipe with a diameter of 5 mm was used as pipeline for gas charging. During the experiment, rapture discs with threshold values of 0.5 MPa, 0.74 MPa and 1.0 MPa were prepared for triggering an outburst. If the value 0.5 MPa failed, the experiment proceeded with a higher value, beginning with the sealing procedure. As the model was completed, cover plates and sealing ring were installed and loading system maintained the stress of 1.5 MPa on the model (local concentration stress 2.4 MPa).

#### Gas charging and completion

The gas-tightness can be tested by dropping some soapy water in connected position after injecting a certain amount of air into chamber. If in good gas-tightness, the chamber will be outgassed to vacuum and gas charging will be started in 12 hours later. Non-explosive carbon dioxide (CO_2_) was used for the experiment. Firstly, CO_2_ with pressure of 0.2 MPa was injected into the chamber repeatedly till the pressure in the chamber was relatively stable. And it was continued with the injection pressure of 0.3 MPa. In order to ensure enough safety margin of rupture disc, the injection was terminated as soon as 0.3 MPa gas pressure was maintained. Finally, the occurrence of an outburst was induced by injecting gas with pressure of more than 0.5 MPa.

## Results

### Variation of gas pressure

**Figure 6:**
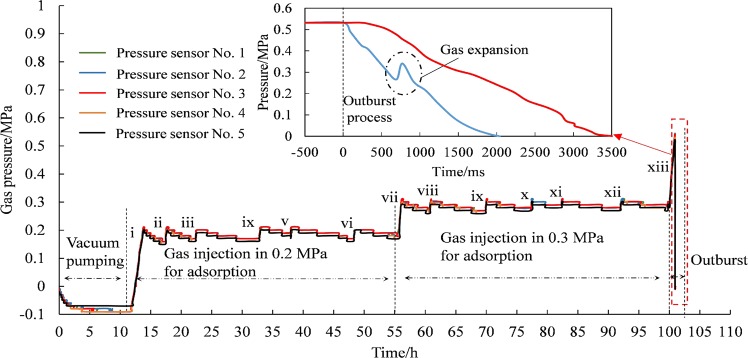
The pressure variation of gas pressure during test (i, ii, iii, iv, v, vi, vii, viii, ix, x, xi, xii, xiii represent the sequence number of gas charging).

Gas pressure was recorded by pressure sensors during the experiment as shown in Fig. 6. As gas pressure remained −0.09 MPa about 6 h after vacuum pumping, the air tightness was in good condition. The gas pressure fluctuated observably in the process of 0.2 MPa and 0.3 MPa gas charging. Injected gas in the chamber was adsorbed constantly by coal sample, resulting in pressure decrease. Repeated gas charging led pressure up and down till pressure stayed stable, approaching adsorption equilibrium. According to characteristic curve of adsorption isotherm, to some extent, gas adsorbed quantity increases as pressure rises^20,21^. Thus, the variation tendency of 0.3 MPa gas injection was similar to that of 0.2 MPa injection.

While the injection pressure increased to 0.53 MPa, over the threshold value of rupture disc tearing, a prominent pressure gradient between coal sample and ambient environment led to the occurrence of outburst. The values recorded by No. 2 and No. 3 sensors dropped to zero respectively in 2 seconds and 3.5 seconds. No. 2 sensor underwent a pressure rise at 700 ms and dropped at 800 ms. The small pressure recovery was due to the sealing effect of coal and rock fragments, and large amount of desorbed gas rapidly expanding to push them outside. The pressure of No. 3 sensor, far from outburst port and closer to inner chamber, declined slowly and lasted longer. It is due to a smaller pressure gradient at this position that leads to a smaller desorption rate.

### Variation of temperature

**Figure 7:**
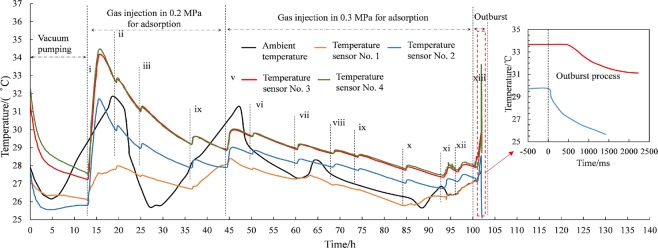
The temperature variation of coal sample during test (i, ii, iii, iv, v, vi, vii, viii, ix, x, xi, xii, xiii represent the sequence number of gas charging).

Temperature variation can influence the process of adsorption and desorption significantly. Adsorption process is exothermic and desorption process endothermic^22,23^. According to the temperature records of sensors during the experiment, the varied tendency of coal sample was consistent with ambient environment, as shown in Fig. 7. Sections of coal sample close to outburst port can exchange heat with outside more efficiently, and the physical process of adsorption and desorption were affected by ambient temperature easily. So the temperature of No. 1 sensor was the lowest and the next was No. 2 sensor. Values of No. 3 and No. 4 sensors were almost the same, not been seriously affected.

At the stage of vacuum pumping the temperature declined rapidly. It carried out 13 times gas charging during experiment, each accompanied by a small increase of temperature and subsequent decrease with ambient temperature down. All sensors’ values reached the maximum in the first gas charging, because coal sample adsorbed large amount of gas at one time. While injection pressure increased to 0.53 MPa, the temperature increased rapidly until the outburst occurred. Then a rapid temperature drop occurred because of a large amount of gas desorption. No. 2 sensor had a high descending rate and a large amplitude, compared with No. 3 sensor. It is located closer to outburst port, where the gas had a larger desorption rate.

### Propagation of outburst coal

**Figure 8:**
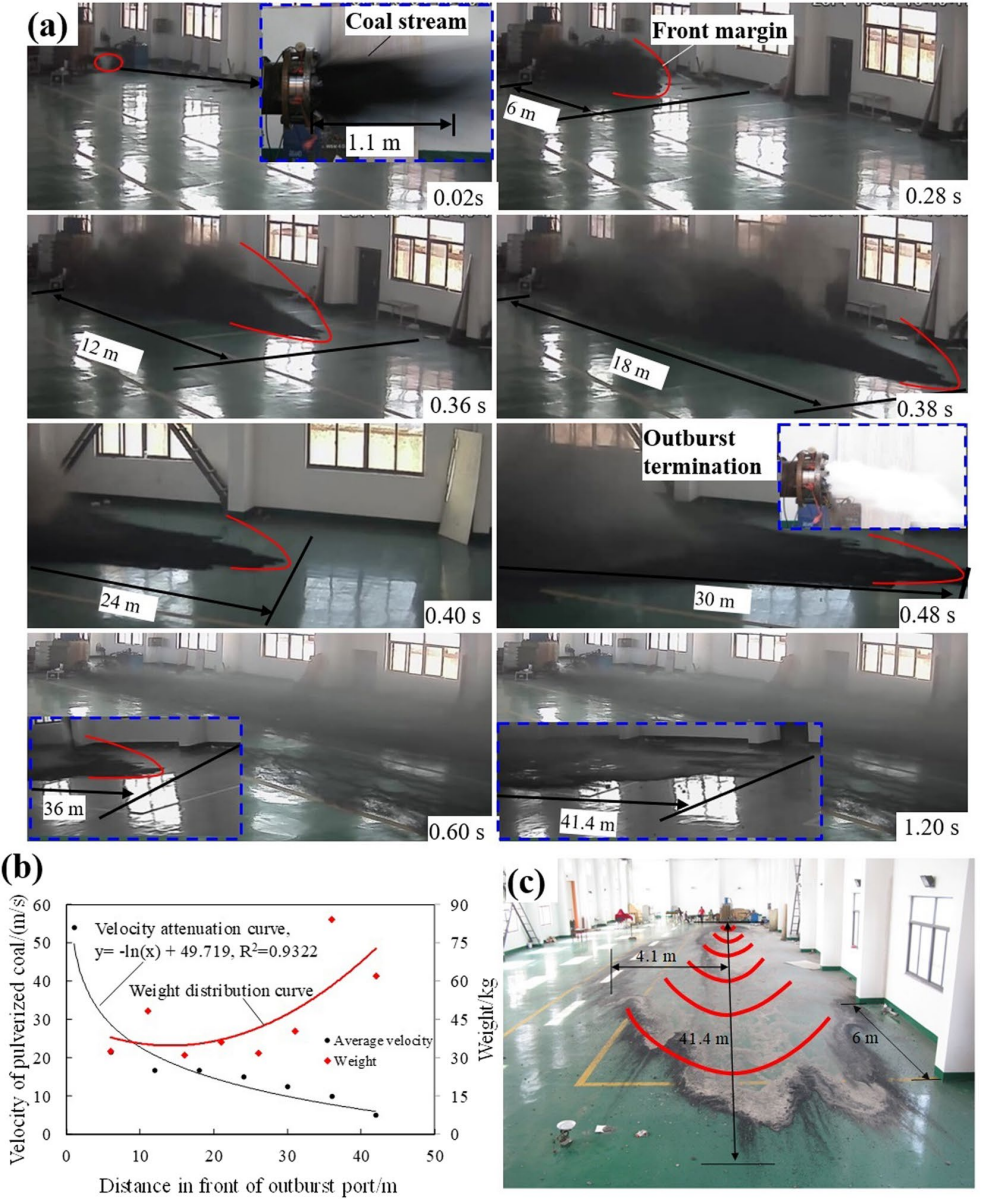
Records about propagation of outburst coal. (**a**) The captured photograph of outburst propagation process (Time stamping is the spacing interval; Length is cumulative propagation distance). (**b**) Variation of outburst propagation velocity. (**c**) Fan-shaped distribution of outburst coal.

The propagation time of the front margin of outburst coal stream were measured by photography as shown in Fig. 8(a). The velocity of coal stream can be calculated by velocity-time formula. Relationship between velocity of outburst coal and propagation distance can be obtained by curve fitting as: y = −11.7ln(x) + 49.719, R^2^ = 0.9322 (Fig. 8(b)). The propagation velocity of coal stream was at high speed and decreased rapidly with the propagation distance increasing. An average velocity of outburst coal approached 21.43 m/s at 1 m to 6 m in front of the outburst port, and it decayed to a minimum of 4.5 m/s at 36 m to 42 m. While pulverized coal did not erupt at 1.92 s, outburst propagation faded. Therefore, the outburst terminated at 1.92 s and outburst propagation lasted 3.7 s. About 369.9 kg specimens were expelled, including coal and rock samples. The expelled materials appeared as fan-shaped distribution, and maximum propagation distance was 41.4 m (Fig. 8(c)).

**Figure 9:**
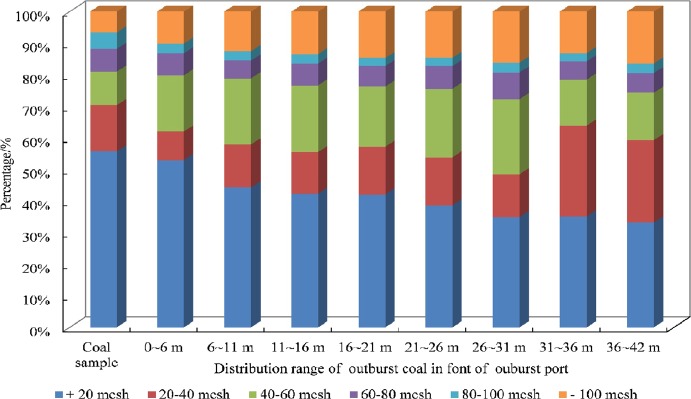
The classification of particle sizes of outburst coal.

The expelled coal was collected and divided into 8 groups, from 0 to 6 m, 6 to 11 m, 11 to 16 m, 16 to 21 m, 21 to 26 m, 26 to 31 m, 31 to 36 m and 36 to 42 m of fan-shaped area in front of the outburst port. The possessed propagation distances of 8 groups were 6 m, 16 m, 21 m, 31 m, 36 m, and 42 m respectively. 8 groups of outburst coal with an open space underwent the similar velocity attenuation process, though at different positions. According to equation *y = *−11.7*ln*(*x*) + 49.719 (Fig. 9B), the velocity attenuation equations of outburst coal can be obtained by modifying dependent variable *x*. The average velocity of the sampled coal can be arithmetic average of initial and final velocities. The equations and calculated results were shown in Table 3. Kinetic energy was the main form of outburst propagation and the velocity of outburst coal was the characteristic parameter that manifest the outburst propagation. It was obtained by a mass-weighted calculation of the velocities of the 8 groups outburst coal (Eq. 3) and the calculated result was 17.21 m/s.

3 $$v={\sum }_{i=1}^{n}\frac{{m}_{i}}{{m}_{total}}{v}_{i}$$

where *v* is the propagation velocity of outburst. *v*_*i*_ is the average velocity of the outburst coal with mass *m*_*i*_ and *n* = 8. *m*_*total*_ is the total mass of sampled coal.

**Table 3:** Velocity calculation according to propagation distance of outburst coal.

ID	Distribution range/m	Propagation distance/m	Velocity attenuation equation	Initial velocity/(m/s)	Final velocity/(m/s)	Average velocity/(m/s)	Weight/kg
1	0~6 m	6	*y* = −11.7*ln*(*x* + 36) + 49.719	7.99	6.16	7.07	32.6
2	6~11 m	11	*y* = −11.7*ln*(*x* + 31) + 49.719	9.77	6.16	7.96	48.3
3	11~16 m	16	*y* = −11.7*ln*(*x* + 26) + 49.719	11.87	6.16	9.01	31.2
4	16~21 m	21	*y* = −11.7*ln*(*x* + 21) + 49.719	14.44	6.16	10.30	36.2
5	21~26 m	26	*y* = −11.7*ln*(*x* + 16) + 49.719	17.73	6.16	11.94	31.8
6	26~31 m	31	*y* = −11.7*ln*(*x* + 11) + 49.719	22.32	6.16	14.24	40.4
7	31~36 m	36	*y* = −11.7*ln*(*x* + 6) + 49.719	29.99	6.16	18.07	84.2
8	36~42 m	41	*y* = −11.7*lnx* + 49.719	76.66	6.16	41.41	62

### Pulverized coal distribution

The outburst coal was sampled and sieved to particles larger than 20 mesh, 20 to 40 mesh, 40 to 60 mesh, 60 to 80 mesh, 80 to 100 mesh and smaller than 100 mesh (Fig. 9). The scattering fragments of coal or rock material was closer to outburst port. The small size grains were far away from outburst port. The weight of particles smaller than 100 mesh increases with the distance in front of the outburst port. Particles larger than 20 mesh possessed a large mass percent in front of outburst port and reached a maximum proportion in a range of 0 to 6 m.

Proportions of particle size fraction of outburst coal had a significant change, compared with original coal sample. Particles of outburst coal smaller than 100 mesh took a higher percentage than coal sample. Correspondingly, particles larger than 20 mesh took a smaller percentage. It is indicated that the small size coal particles tend to participate in the outburst process. This can be interpreted as result of coal particles with small sizes possessing a higher desorption rate^24^. They originate from the original coal sample and the breakdown of coal in process of the outburst.

### Energy analysis about experiment

The process of an outburst obeys the energy conservation law. The elastic strain energy (*E*_*e*_), gas expansion energy (*E*_*p*_) and internal energy of coal (*ΔU*) convert into the breakage work (*W*_1_), throwing out work (*W*_2_) and gas-flow loss energy (*ΔE*). They can be expressed as follows:

4 $${E}_{e}+{E}_{p}+\Delta U={W}_{1}+{W}_{2}+\Delta E$$

The strain energy stored in the coal mass under the effect of stress can be expressed as follows:

5 $${E}_{e}=\frac{1}{2E\rho }[{\sigma }_{1}^{2}+{\sigma }_{2}^{2}+{\sigma }_{3}^{2}-2\mu ({\sigma }_{1}{\sigma }_{2}+{\sigma }_{2}{\sigma }_{3}+{\sigma }_{1}{\sigma }_{3})]$$

where *σ*_1_, *σ*_2_ and *σ*_3_ are the three principal stresses. *ρ* is the density of coal and *E* is the Young’s modulus. *V* is the volume of coal mass and *μ* is the Poisson’s ratio. Considering the apparatus providing full constraint at zero displacement in horizontal direction, the horizontal stress can be calculated by *σ*_2_ = *σ*_3_ = *σ*_1_ * μ/(1 − *μ*).

Gas expansion energy stored by free gas can be expressed as follows^25^:

6 $${E}_{P}=\frac{{p}_{1}{V}_{1}}{\gamma -1}[{(\frac{{p}_{0}}{{p}_{1}})}^{\frac{\gamma -1}{\gamma }}-1]$$

where *p*_0_ and *p*_1_ are gas pressure and atmospheric pressure; *V*_1_ is the volume of gas emission; *γ* is the process index. For an isothermal process, *γ* = 1, for an adiabatic process, *γ* = 1.31, and for a changeable process, *γ* = 1~1.31.

Gas stored in coal mass can be divided into free gas and absorbed gas. In the process of outburst, the gas emission volume *V*_1_ contains free gas and desorbing gas and can be expressed by Eq. (5). The free gas is relatively stable and stored in pore space. The desorbing gas increased sharply with the initiation of outburst till the end of outburst. The content of free gas and absorbed gas in coal mass can be calculated by Eqs (8) and (9)^5,26^.

7 $${V}_{1}={\alpha }_{t}({X}_{1}-{X}_{1}^{^{\prime} })+{X}_{2}$$

8 $${X}_{1}=\frac{ab{p}_{0}}{1+b{p}_{0}}\times \frac{1}{1+0{\rm{.31}}{M}_{ad}}{e}^{n({T}_{s}-{T}_{0})}\times \frac{100-{M}_{ad}-{A}_{d}}{100}$$

9 $${X}_{2}=\frac{{V}_{0}{p}_{1}T}{{T}_{0}{p}_{0}\zeta }$$

where *X*_1_ and *X*_2_ are respectively the content of absorbed gas and free gas; *X*_1_′ *is* the content of absorbed gas at atmospheric pressure; *α*_t_ is the desorption rate of coal particle at time *t*; *T*_s_ is temperature of the isothermal adsorption test; *T*_0_ and *T* are the temperature respectively before and after outburst; *a* and *b* are the Langmuir adsorption constant (for CO_2_); *V*_0_ is total pore volume per ton of coal; *ζ* and *n* are coefficients that depend on the gas pressure of coal seam.

Based on the diffusion model of Fick, the desorption rate of coal particles can be expressed as Eq. (10)^24,27^.

10 $${\alpha }_{t}=\frac{12}{d}{(Dt/\pi )}^{\frac{1}{2}}$$

where *D* is diffusion coefficient. *d* is the size of coal particle after outburst and can be calculated by Eq. (11).

11 $$d={\sum }_{j=1}^{m}{w}_{j}{d}_{j}$$

where *d* is the mean particle size (m), *w*_*j*_ is the percentage of particles at a diameter of *d*_*j*_ and *m* = 6.

By combining Eq. (6)~(11) the gas expansion energy (*E*_*p*_) can be calculated.

The coal samples undergo the variation of temperature in the process of outburst and internal energy can be calculated by Eq. (12).

12 $$\Delta U=\beta \cdot cm\Delta T$$

where *c* is the heat capacity of coal mass and *ΔT* is the temperature difference before and after outburst*. β* is the uniformity coefficient, 0.5~1. *m* is the mass of outburst coal.

Based on energy consumed per surface area, a formula to calculate breakage work per kilo (*w*_1_) is as follows^25^.

13 $${w}_{1}=\frac{3A\omega }{\rho }(\frac{1}{{d}_{i}}-\frac{1}{{d}_{0}})$$

where *A* is the energy consumed per increasing unit of surface area and *d*_0_ and *d*_*i*_ are mean particle size of coal sample and outburst coal in different areas respectively. *ω* is the uniform coefficient of 1.2~1.7.

According the variation of weight and particle size of outburst material at different position, the weighting breakage work per kilo outburst coal can be calculated by Eq. 12.

14 $${W}_{1}=\frac{3A\omega }{\rho }{\sum }_{i=1}^{n}\frac{{m}_{i}}{m}\cdot (\frac{1}{{d}_{i}}-\frac{1}{{d}_{0}})$$

Kinetic energy of outburst coal can be calculated by equation as follows.

15 $${{\rm{W}}}_{2}=\frac{1}{2}{\sum }_{i=1}^{n}{m}_{i}{v}_{i}^{2}$$

Gas-flow loss energy is the gas kinetic energy while gas phase and coal phase are separated. It can be calculated theoretically by Eq. (16).

16 $$\Delta E=\frac{1}{2}{V}_{1}\rho ^{\prime} {v^{\prime} }^{2}$$

where *ρ*′ and *v*′ are the gas flow density and velocity after gas phase and coal phase are separated. The gas-flow loss energy cannot be calculated directly, because the outburst space is open and the flow field is too complicated to trace. It can be calculated indirectly by Eq. (17).

17 $$\Delta E={E}_{e}+{E}_{p}+\Delta U-{W}_{1}-{W}_{2}$$

The parameters required for energy transformation calculating in the outburst experiment are listed in Table 4 and the calculated results are shown in Table 5. Variation of internal energy of coal (*ΔU*) occurred in the process of gas desorption, as described in Section Variation of temperature. The internal energy (*ΔU*) contributes to desorption behavior of outburst coal. Gas expansion energy (*E*_*p*_) provides the kinetic energy for outburst coal. Thus, gas potential energy, including internal energy and gas expansion energy, is the energy stored in coal due to gas desorption and gas expansion. Gas potential energy took a large percent of initiation energy and were far greater than the coal strain energy (*E*_*e*_). It showed better consistency with the conclusion that the most energy contributing to outburst derived from gas potential energy^24,30^. 31% of total energy was used as breakage work (*W*_1_) and 17% was used to expel material (*W*_2_). Gas-flow loss energy (*ΔE*), participating in the process of an outburst, made up 52% of total energy.

**Table 4:** Parameters for the outburst experiment.

Parameters	Value	Parameters	Value
Principal stress, σ_1_(MPa)	2.4	Temperature of the isothermal adsorption test, *T*_*s*_(K)	298.15
Principal stress, σ_2_(MPa)	1	Temperature of before outburst, *T*_0_(K)	298.15
Principal stress, σ_3_(MPa)	1	Temperature of after outburst, *T*(K)	298.15
Young’s modulus, *E*(MPa)	50	Pore volume, *V*_0_(m^3^/t)	0.1054
Poisson’s ratio, *μ*	0.3	Coefficient about gas pressure, *ζ*	1
Gas pressure, *p*_0_(MPa)	0.3	Coefficient about gas pressure, *n*	1
Atmospheric pressure, *p*_1_(MPa)	0.1	Particle size before outburst, *d*_0_(mm)	0.71
Process index, *γ*	1.3	Particle size after outburst, *d*_1_*, d*_2_*, d*_3_*, d*_4_*, d*_5_*, d*_6_*, d*_7_*, d*_8_, (mm)	0.68, 0.63,0.61,0.61, 0.59,0.56, 0.62, 0.59
Langmuir adsorption constant, *a*(m^3^/t)	31.72	Diffusion coefficient, *D*(mm^2^/s)	0.0007^28^
Langmuir adsorption constant, *b*(MPa^−1^)	1.22	Heat capacity, *c*[kJ/(kg·K)]	0.79^29^
Content of absorbed gas, *X*_1_(m^3^/t)	5.25	Temperature difference, *ΔT*(K)	2
Content of free gas, *X*_2_(m^3^/t)	0.03	Uniformity coefficient, *β*	0.7
Content of absorbed gas at atmospheric pressure, *X*_1_′(m^3^/t)	1.74	Energy consuming Per surface area adding, *A*(J/m^2^)	505
Desorption rate of coal particle, *α*_*t*_	0.38	Uniform coefficient, β	1.5
Gas emission volume, *V*_1_(m^3^/t)	1.36	Outburst propagation velocity, *v*(m/s)	17.21
Average velocity of outburst coal at different positions, *v*_*1*,_ *v*_*2*,_ *v*_*3*,_ *v*_*4*,_ *v*_*5*,_ *v*_*6*,_ *v*_*7*,_ *v*_8_(m/s)	7.07, 7.96, 9.01, 10.30, 11.94, 14.24, 18.07, 41.41	Outburst coal mass at different positions, *m*_*1*,_ *m*_*2*,_ *m*_*3*,_ *m*_*4*,_ *m*_*5*,_ *m*_*6*,_ *m*_*7*,_ *m*_8_(kg)	32.6, 48.3, 31.2, 36.2, 31.8, 40.4, 84.2, 62

**Table 5:** The calculated results of energy.

Energy	*E*_*e*_(J/kg)	*E*_*p*_(J/kg)	*ΔU*(J/kg)	*W*_1_(J/kg)	W_2_(J/kg)	*ΔE*(J/kg)	Total energy(J/kg)
Value	30	171	1060	392	215	654	1261

## Discussion

It took about 25 days to conduct an outburst from debugging experimental system to completing the experiment. A large amount of coal was sampled for an experiment. It was critical to make sure sufficient gas was adsorbed by coal samples. Injecting gas repetitively to specimen by pre-buried pipeline performed well during the experiment. Large amount of coal and gas participated and erupted in the artificial outburst.

The gas content and pressure of coal seam were respectively used as an outburst risk index in many coal enterprises and institutes. Gas content thresholds of 9 m^3^/t for CH_4_ was used in the Sydney Basin to indicate outburst-prone conditions^31^. A critical value of 0.74 MPa gas pressure was widely used to access the risk of outbursts in China^32,33^. And yet some regions in low gas content and pressure can initiate an outburst, known as low-threshold outburst. The statistics of the outburst accidents in Xinmi mining area of China from November 1989 to 2011 showed that 57% of outbursts were with gas content of less than 8 m^3^/t and 75% were with gas pressure of less than 0.74 MPa^34^.

During the experiment, an artificial outburst was conducted with gas content and pressure respectively at 5.28 m^3^/t and 0.5 MPa. Large amount of energy was consumed in coal breakage and throwing out. The coal strength prevented the outburst and a dynamic threshold of gas pressure existed corresponding different damage states of coal^15^. Thus, an outburst corresponds to a type of initial geological states, which may be the low-strength coal, the high gas pressure or gas content and others. The threshold value of an outburst indicator is not stable in coal basins or even different mining area. Coal with the low-strength resistance to an outburst may lead a low-threshold outburst. It is due to the fact that enough energy can pulverize coal and throw it out, especially the gas potential energy.

## Conclusion

An experimental system for the outburst simulation on a middle scale was composed of test chamber, distributed loading system, outburst inducing device, vacuum pumping and gas charging system, and data acquisition system. Characterization of gas pressure and temperature variation, outburst coal propagation, pulverized coal distribution and energy consuming were investigated based on an artificial outburst. According to the results obtained, some conclusions can be made:

1. The experimental system can simulate field prototype outburst at a one-meter scale, but have some limitations, such as one-dimensional loading and gas pressure less than 2 MPa.
2. An outburst is influenced by gas pressure and temperature significantly, because of the coal behavior of adsorption and desorption. The dynamic effects of outburst can be analyzed by the propagation of outburst coal and the proportions of pulverized coal.
3. The calculated energy results show that gas potential energy takes a large percent of initiation energy and gas-flow loss energy occupies the greatest proportion of energy consuming. 31% of the energy was used as breakage work while 17% of the energy was used to expel the material.
4. The outburst conducted with 0.5 MPa gas pressure and 5.28 m^3^/t gas content was called a low-threshold outburst. It is a common phenomenon and should be taken seriously in underground mining. The gas potential energy can be the main factor that pulverize the low strength coal and throw it out.
